# Corrigendum to: Huperzine A ameliorates neurological deficits after spontaneous subarachnoid hemorrhage through endothelial cell pyroptosis inhibition

**DOI:** 10.3724/abbs.2024067

**Published:** 2024-05-06

**Authors:** Qiang Hu, Rong Zhang, Xiaoqiao Dong, Dingbo Yang, Quan Du, Wenhua Yu

*Acta Biochimica et Biophysica Sinica*, 2024 Mar 26; 56(4): 645–656

https://doi.org/10.3724/abbs.2024037

In the original version of this manuscript, errors were found in authors and affiliations. Modified authors and affiliations are as follows. The authors apologize for the errors.

Qiang Hu ^1,2,†^, Rong Zhang ^3,†^, Xiaoqiao Dong ^1,2^, Dingbo Yang ^1,2^, Quan Du ^1,2,^* and Wenhua Yu ^1,2,^*

^1^Department of Neurosurgery, The Affiliated Hangzhou Hospital of Nanjing Medical University, Hangzhou 310000, China, ^2^Department of Neurosurgery, Affiliated Hangzhou First People’s Hospital, School of Medicine, Westlake University, Hangzhou 310000, China, and ^3^Medical Examination Center, Affiliated Hangzhou First People’s Hospital, School of Medicine, Westlake University, Hangzhou 310000, China

^†^These authors contributed equally to this work.

*Correspondence address. Tel: +86-571-56005600; E-mail: duquan76@zuaa.zju.edu.cn (Q.D.) / E-mail: ywh669@126.com (W.Y.)

